# Remembering the Object You Fear: Brain Potentials during Recognition of Spiders in Spider-Fearful Individuals

**DOI:** 10.1371/journal.pone.0109537

**Published:** 2014-10-08

**Authors:** Jaroslaw M. Michalowski, Mathias Weymar, Alfons O. Hamm

**Affiliations:** 1 Department of Biological and Clinical Psychology, University of Greifswald, Greifswald, Germany; 2 Faculty of Psychology, University of Warsaw, Warszawa, Poland; University of Medicine & Dentistry of NJ - New Jersey Medical School, United States of America

## Abstract

In the present study we investigated long-term memory for unpleasant, neutral and spider pictures in 15 spider-fearful and 15 non-fearful control individuals using behavioral and electrophysiological measures. During the initial (incidental) encoding, pictures were passively viewed in three separate blocks and were subsequently rated for valence and arousal. A recognition memory task was performed one week later in which old and new unpleasant, neutral and spider pictures were presented. Replicating previous results, we found enhanced memory performance and higher confidence ratings for unpleasant when compared to neutral materials in both animal fearful individuals and controls. When compared to controls high animal fearful individuals also showed a tendency towards better memory accuracy and significantly higher confidence during recognition of spider pictures, suggesting that memory of objects prompting specific fear is also facilitated in fearful individuals. In line, spider-fearful but not control participants responded with larger ERP positivity for correctly recognized old when compared to correctly rejected new spider pictures, thus showing the same effects in the neural signature of emotional memory for feared objects that were already discovered for other emotional materials. The increased fear memory for phobic materials observed in the present study in spider-fearful individuals might result in an enhanced fear response and reinforce negative beliefs aggravating anxiety symptomatology and hindering recovery.

## Introduction

Individuals suffering from specific phobias exhibit an excessive and unreasonable fear of their phobia-relevant objects or feared situations. Moreover, phobic individuals detect even minor signals of upcoming threat at a very early processing stage [1]–[2]. When the threat cue does not disappear or even approaches, this increased attention is followed by defensive response mobilization, as indexed by cardiac acceleration and startle potentiation [3] to prepare the organism for effective escape if possible.

It is well established that our survival depends not only on the ability to activate such functional behavioral adjustments to threatening situations but also to increase the chance that survival-relevant information is available in the future [4]. In fact, multiple evidence suggest that emotionally arousing events are better remembered than affectively neutral events (for review see [5]–[6]) as demonstrated in studies using free recall [7] and recognition memory procedures [8]–[10]. The question arises whether mnemonic processing of feared objects also varies with inter-individual fear status? One might expect that individuals with specific phobia show better memory of their feared objects due to stronger emotional arousal elicited by these events. Previous studies, however, have found mixed results. One Positron Emission Tomography (PET) study [11] found better memory discrimination for phobic pictures compared to non-phobic pictures in participants with animal phobia. Moreover, in this study the memory performance covaried with amygdala activation and electrodermal activity during encoding, supporting the arousal hypothesis [5]–[6]. In contrast, studies using words as stimuli did not find memory enhancing effects for phobia-relevant words in explicit memory tests such as recognition or recall [12]–[14]. For instance, memory recall for a spider word presented in a continuous stream of neutral pictures did not differ between spider phobics and non-phobic controls [12]. In another study, spider phobic participants recalled fewer spider related words (e.g., cobweb, fangs) compared to neutral words, in the presence of a live spider [14] Similarly, Thorpe and Salkovskis [13] observed that individuals with spider phobia did not differ from non-fearful controls in their recognition memory (hits and false alarms) for live spiders presented in video clips. Moreover, even poorer recognition memory for big dead spiders was observed for individuals with animal phobia compared to non-phobic participants in the study by Watts, Trezise, and Sharrock [15].

Several methodological issues might have contributed to these inconsistent findings: First, phobic stimulation might engage an avoidance tendency, as postulated in the attention-avoidance hypothesis [16], and/or overload the encoding capacities interfering with subsequent detailed processing of phobia-relevant information. In fact, previous eye movement data showed that individuals with specific phobia tend to avoid a detailed perceptual analysis of their feared objects after an increased initial orienting [17]–[18]. Second, the poorer quality of cognitive representations and reduced memory performance for phobic stimuli might result from short intervals between initial stimulus presentation and the memory test. A number of studies revealed that the storage of emotional material benefits from longer consolidation intervals [6], [19]–[21]. Using longer time intervals resulted in a higher memory accuracy and recollective experience for emotionally arousing relative to neutral events when compared to short (immediate) time lags. Third, arousal levels of the spider material could vary across the different experiments. For instance, arousal levels are lower for words than for affective pictures in general [22]–[23], thus pictorial materials of spiders may be more effective in prompting emotional arousal [11] than spider-related words leading to better memory performance for pictures than for words.

Thus, in the present study, we used pictorial scenes, a longer memory consolidation interval (1-week), and increased the number of repetitions during encoding to counteract shallow encoding due to possible avoidance tendencies engaged by phobic stimulation. In addition, we investigated recognition memory using Event-Related Potentials (ERPs), which provides a more direct insight into memory processing mechanisms [24]. Numerous studies reported differences in the ERP waveform between items presented for the first time and repeated items (for review see [25]). Specifically, during recognition, correctly recognized old stimuli reliably elicited a more positive-going ERP deflection than correctly classified new stimuli. An early frontal old/new difference between 300 and 500 ms has been linked to familiarity-based recognition [26] and a later occurring (∼400–800 ms) centro-parietal old/new effect was suggested to index successful recollection of information [27]–[29]. Importantly, previous ERP studies found that this later centro-parietal old/new effect is more pronounced for emotional, relative to neutral words [30], [31], facial expressions [32] and natural scenes [23], indicating that better recognition of emotional events is related to explicit recollection [6].

In the present study we presented unpleasant, neutral and spider pictures to spider-fearful and control individuals and tested recognition memory for these materials. Considering previous evidence suggesting that the storage of emotional material benefit from longer consolidation intervals [6], [19]–[21], we used a delay of 7 days between encoding and memory test. Moreover, given the high homogeneity of phobia-relevant spider pictures these materials are more difficult to memorize when compared to images depicting neutral and unpleasant scenes. In order to overcome this problem and to ensure that there is an appropriate amount of trials for analyzing EPRs we included photographs depicting different exemplars of spiders and increased initial picture presentation time and frequency. Accordingly, during encoding session, each picture was presented three times to ensure deeper encoding. We expected to replicate previous findings of better recognition memory and larger ERP old/new difference for emotional (unpleasant and spider pictures) pictures, compared to neutral pictures. Moreover, if spiders induce more emotional arousal in spider-fearful individuals than in controls, memory for spider pictures should be better in the high fearful relative to the control group. Previous ERP studies [1], [33]–[35] observed facilitated perceptual processing of spider pictures in spider-fearful relative to control individuals presumably in the P1, Early Posterior Negativity (EPN), and Late Positive Potentials (LPP). We also tested whether such ERP differences occur during recognition when old and pictures are presented.

## Materials and Methods

### Participants

31 students from the University of Greifswald participated in two study sessions (encoding and recognition). Participants were selected from a pool of 532 students from the University of Greifswald who were screened with the German version of the 31-item spider phobia questionnaire (SPQ; German version, [36]). 16 participants (15 females) scoring above the 85^th^ percentile of the distribution on the SPQ were included in the spider-fearful group (M = 19.7, SD = 3.1) and 15 female participants scoring below the 33^th^ percentile of the distribution were included in the non-fearful control group (M = 3.5, SD = 1.4). The groups did not differ in general anxiety as measured by the State-Trait Anxiety Inventory (STAI-T, [37]), t (28)<1, ns. Participants received either course credit or 24 Euros for participation. The study protocol was approved by the Research Ethics Committee of the Faculty of Psychology University of Warsaw. All subjects gave their written informed consent. Data from one female spider-fearful participant were excluded from further analyses because of excessive EEG artifacts.

### Stimulus Materials and procedure

Overall, 256 color photographs were selected from the International Affective Picture System (IAPS; [38]) and from our own picture pool (see [39]–[42]). The pictures included 128 neutral (e.g., landscapes, buildings and neutral people), 64 unpleasant (e.g., mutilation, human and animal attack), and 64 fear-relevant pictures of spiders.

**Figure 1:**
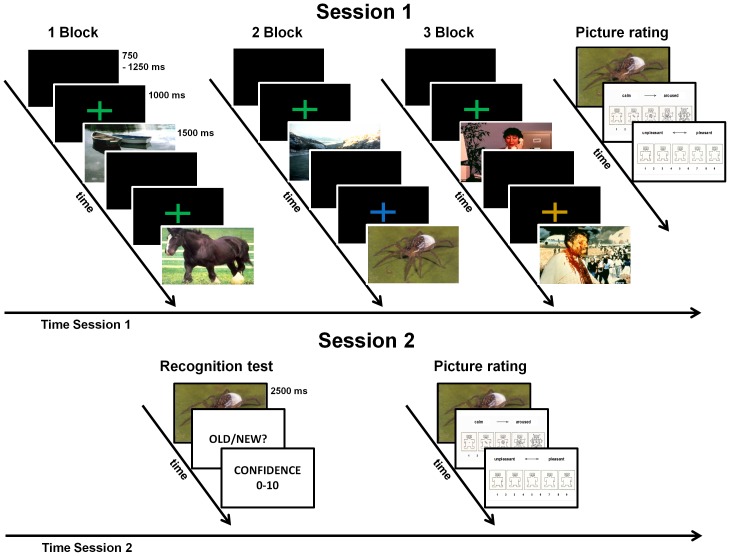
Illustration of the experimental procedure. In the first session (top) 3 separate picture blocks (neutral, spider and neutral, unpleasant and neutral) were presented in a randomized order. Within each block pictures were preceded by a fixation cross in one of three different colors that signaled the category of an upcoming picture. At the end of this session pictures were rated for valence and arousal. In the second session (bottom) old and new pictures were presented and participants were asked to decide for each picture whether it has been presented before in the study or not (OLD/NEW?) and to rate their confidence (0–10). Finally, new pictures were rated for valence and arousal.

During the encoding session, participants viewed a set of 160 pictures presented within three separate blocks: 32 neutral pictures (block 1), 32 neutral intermixed with 32 spider pictures (block 2), and 32 neutral intermixed with 32 unpleasant pictures (block 3, see Figure 1). The block order was counterbalanced across participants. In each block pictures were presented twice in a pseudo-random order with the restriction that the same picture could not occur on two consecutive trials. Each picture was presented for 1500 ms, preceded by a fixation cross (1000 ms) and followed by an intertrial interval (ITI) of 750, 1000, or 1250 ms (in random order). The color of the fixation cross (blue or green or dark yellow equated in brightness) signaled the category of the upcoming picture. Assignment of colors to the specific picture category (neutral, unpleasant, spider) was counterbalanced across subjects. A cue signaling neutral pictures remained the same across all three experimental blocks. At the end of the session each participant was asked to view each picture as long as desired and to press a button to terminate picture presentation. After each picture offset valence and arousal ratings were collected using a computerized version of the Self-Assessment Manikin [43]. During the encoding session, no mention of a memory test was made (incidental encoding). These first session ERP data are reported elsewhere.

One week after the encoding session, 96 old and 96 new pictures (32 neutral, 32 unpleasant, and 32 spider pictures, respectively) were presented for 2500 ms and participants were instructed to decide for each picture whether it has been presented before in the study by pressing a button marked “yes” or “no” after picture offset. Following the recognition decision, participants rated the recognition confidence (see [10], [44]) on a 11-point Likert scale (0 – not confident, 10 – absolutely confident). At the end of the recognition session the EEG sensors were removed and valence and arousal ratings for all new pictures were obtained from the participants using the Self-Assessment Manikin [43]. During both sessions participants were seated in a recliner in a dimly lit and sound-attenuated room in front of a 20-inch (50.8 cm) computer monitor located approximately 1.5 m from their eyes (11° of visual angle).

### Data acquisition, recording and reduction

Electrophysiological data were collected from the scalp using a 256-sensor net (Electrical Geodesics, Inc., Eugene, OR). Electrode impedance was kept below 30 kΩ as recommended by the manufacturer. EEG data were continuously recorded with the vertex sensor as a reference electrode, in the 0.1–100 Hz frequency range with a sampling rate of 250 Hz. Continuous electroencephalography (EEG) data were low pass filtered at 40 Hz using digital filtering before stimulus synchronized epochs lasting from −120 ms to 1000 ms relative to the picture onset were extracted. Data editing and artifact rejection were based on a method for statistical control of artifacts [45]. Artifact rejection was based upon boundary values of three parameters: maximal absolute value over time, standard deviation over time, and maximal temporal gradient over time. First, the data with common (vertex) reference were used to detect and reject channels with artifacts. Eye movement and blink artifacts were reduced using a regression-based procedure as implemented in BioSig [46]. Second, the data were transformed to averaged reference and global artifacts were detected and contaminated trials excluded from further analysis. Overall, approximately 25% of the trials were rejected because of artifacts. The rejected trials were equally distributed across picture categories and groups. For the remaining trials, rejected single channels were estimated by a spherical spline interpolation on the basis of all remaining sensors on a trial-by-trial base. Data reported are baseline-corrected and converted to an average reference.

### Statistical data analysis

#### Behavioral Data

Hit rate (H), false alarm (FA), recognition accuracy (Pr (p (hit)−p (false alarms)) and response bias Br (p (false alarms)/p (1−Pr)) were analyzed in the recognition task. According to Snodgrass and Corwin [47] greater Pr values indicate better discrimination between old and new items. Br values higher than 0.5 indicate liberal response criteria (bias to respond “old”) and lower than 0.5 suggest conservative response criteria (bias to respond “new”). These behavioral performance measures were analyzed with repeated measures analysis of variance (ANOVA) including Picture Category (neutral vs., unpleasant vs., phobia-relevant) as a within-subject factor and Group (spider fear vs., control) as a between-subject factor.

Confidence ratings were analyzed with repeated measures ANOVAs including Memory (old vs. new) and Picture Category as within-subject factors as well as Group as a between-subject factor. Valence and arousal ratings as well as the viewing time were analyzed separately using an ANOVA involving Group as a between-subject factors and Picture Category as a within-subject factor.

#### Event-Related Potentials

**Figure 2:**
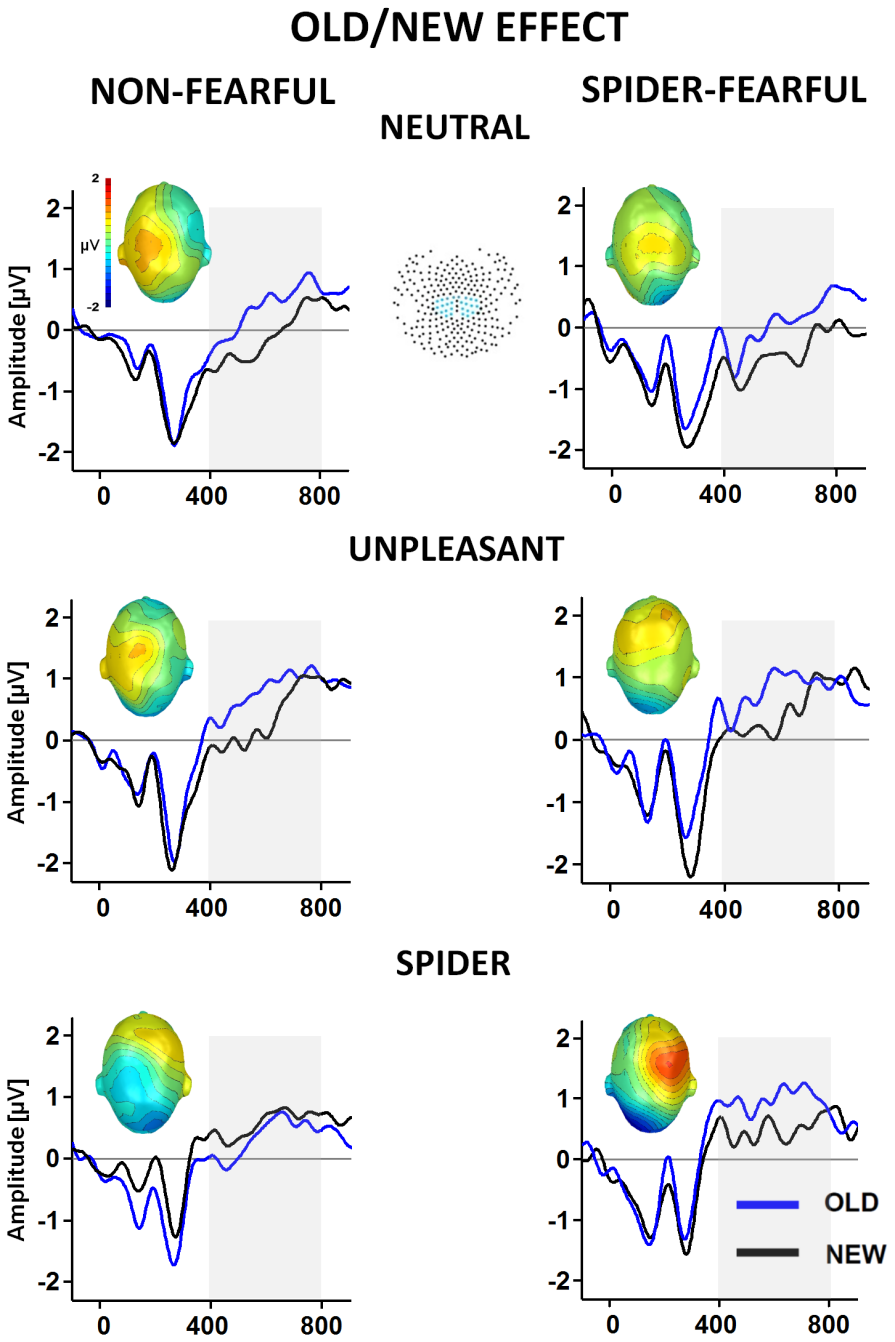
ERP old/new effect in non-fearful and spider-fearful individuals exposed to pictures of naturalistic scenes. The Figure highlights ERP waveforms averaged across centro-parietal channel cluster (see inlet) elicited by correctly classified old and new neutral, unpleasant and spider pictures as well as topographical difference maps (old minus new) displayed for non-fearful control (left) and spider-fearful individuals (right). Shaded areas mark the time interval 400–800 ms selected for the analysis of the ERP old/new effect.

As in previous studies [10], [48], visual inspection of the ERP waveforms as well as single-sensor waveform analyses were used in concert to identify the temporal and spatial characteristics of the old/new ERP effects. For the single-sensor waveform analyses, repeated measures ANOVAs including the within-factors Picture Category (neutral vs., unpleasant vs., phobia-relevant) and Memory (old vs., new) as well as the between-factor Group (spider fear vs., control) were carried out for each time point after picture onset and each individual sensor (cf. [48]). To avoid false positives and to ensure a more stringent alpha-level adjustment, significant effects were only considered meaningful when observed for at least eight continuous data points (32 ms) and two neighboring sensors. These analyses revealed differences in the ERP waveforms for correctly recognized old and new pictures as well as the effects of emotional ERP modulation. For detailed analyses of these effects, mean amplitudes averaged within time windows and sensor clusters identified by both visual inspection and single-sensor waveform analyses were included in further statistical analyses. The ERP old/new effect and the emotional LPP modulation were analyzed within the time window from 400 to 800 ms after picture onset in two central sensor clusters comprising the following sensors: 9, 44, 45, 52, 53, 59, 60, 66, 78, 79, 80, 88, 89 in the left hemisphere and 130, 131, 132, 142, 143, 144, 154, 155, 164, 183, 184, 185, 186 in the right hemisphere (see inlet in Figure 2). The P1 was scored in the time window 124–172 ms in two posterior sensor clusters including 96, 97, 98, 106, 107, 108, 115, 116, 117, 124, 125 in the left hemisphere and 138, 139, 149, 150, 151, 152, 159, 160, 161, 169, 170 in the right hemisphere. The EPN was scored in the time window 200–300 ms for two posterior sensor clusters: 114, 115, 116, 121, 122, 123, 124, 133, 134, 135, 136 in the left hemisphere and 148, 149, 150, 157, 158, 159, 166, 167, 168, 174, 175 in the right hemisphere. Further analyses were carried out by calculating repeated measures ANOVAs including Picture Category, Memory, and Laterality (right vs. left) as within factors and Group as a between factor. Follow-up ANOVAs were calculated for each picture category including Memory (old vs., new) and Laterality (right vs., left) as within factors and Group as a between factor. In order to analyze the emotional ERP modulation, we further compared emotional (spider or unpleasant pictures) with neutral picture contents. For effects involving repeated measures, the Greenhouse-Geisser correction of degrees of freedom was applied.

## Results

### Behavioral Data

#### Ratings and viewing times

Hedonic valence and arousal ratings corresponded with the IAPS norms [38]. When compared to neutral images pictures depicting unpleasant scenes and spiders were rated as more unpleasant, Picture Category, F (1.691, 56) = 262.37, p<.001, η2 = .90, and more arousing, Picture Category, F (1.867, 56) = 196.20, p<.001, η2 = .87. These differences were modulated by group, Picture Category×Group, Fs>31, ps<.001. As expected, individuals with spider fear rated pictures of spiders as more arousing and more unpleasant than control subjects, ts (28)>6, ps<.001. The two experimental groups did not differ in the arousal and valence ratings for neutral pictures, ts (28)<1.3, ns, and in the arousal ratings for unpleasant pictures, ts (28)<1.3, ns. The latter picture category was rated as more unpleasant in the spider fear when compared to the control group, t (28) = 2.2, p<.05. Similar viewing times were found for the three picture categories, Picture Category, F (1.538, 56) = 2.88, p = .080, η2 = .09, Picture Category×Group, F (1.538, 56) = .97, p = .367, η2 = .03. Follow-up tests revealed shorter viewing durations of spider pictures in spider-fearful than control individuals, t (28) = 2.2, p<.05, and no group differences in the viewing time for the two other picture categories, ts (28)<1.3, ns.

#### Memory performance

Table 1 shows the memory performance data (hits, false alarms, discrimination index, response bias and mean confidence ratings) as a function of picture category and group. As expected, hit rates varied as a function of Picture Category, F (1.274, 56) = 49.03, p<.001, η2 = .64. Replicating previous findings, unpleasant pictures were better remembered than neutral pictures, F (1, 28) = 8.35, p<.01, η2 = .23. Unpleasant, F (1, 28) = 66.98, p<.001, η2 = .71, but also neutral pictures, F (1, 28) = 40.66, p<.001, η2 = .59, were better recognized than pictures depicting spiders. Hit rates did not differ between groups, Picture Category×Group, F (1.274, 56)<1, ns. Statistical analyses calculated for false alarms rates resulted in significant effect of Picture Category, F (1.539, 56) = 34.70, p<.001, η2 = .55, and the interaction Group×Picture Category showed a trend towards significance, F (1.539, 56) = 3.29, p = .059, η2 = .11. False alarms rates were lower for unpleasant when compared to neutral pictures, F (1, 28) = 39.26, p<.001, η2 = .58, an effect that did not differ between both groups, Picture Category×Group, F (1, 28)<1, ns. Moreover, there were significantly higher false alarms rates for spider than for unpleasant pictures, F (1, 28) = 54.02, p<.001, η2 = .66, and neutral pictures, F (1, 28) = 13.40, p<.01, η2 = .32. The discrimination index also differed as a function of Picture Category, F (1.363, 56) = 116.46, p<.001, η2 = .81. Better discrimination was observed for unpleasant than neutral and spider pictures, F (1, 28) = 60.77, p<.001, η2 = .68, and F (1, 28) = 172.04, p<.001, η2 = .86, respectively. Moreover, the discriminability was significantly poorer for spider when compared to neutral pictures, F (1, 28) = 78.17, p<.001, η2 = .74. Overall ANOVAs did not show differences in the response bias between the three different picture categories, F (1.795, 56) = 1.73, ns. Although discrimination index Pr was higher and response bias Br was more conservative for spiders in spider-fearful participants (see Table 1), Pr and Br did not differ as a function of Group, Fs (1, 28)<1.3, ns, or Picture Category×Group, F (1.363, 28) = 1.07, ns and F (1.795, 28) = .62, ns for Pr and Br, respectively.

**Table 1:** Behavioral data.

	H	FA	Pr	Br	Confidence	Confidence
					(Old)	(New)
**Unpleasant**						
Control	.93 (.07)	.05 (.06)	.88 (.09)	.45 (.41)	9.4 (.57)	8.8 (.73)
Spider Fear	.94 (.07)	.06 (.08)	.88 (.09)	.54 (.38)	9.4 (.65)	8.9 (.72)
**Neutral**						
Control	.89 (.10)	.13 (.08)	.76 (.11)	.57 (.32)	9.0 (.77)	7.5 (1.10)
Spider Fear	.90 (.08)	.14 (.12)	.76 (.13)	.60 (.30)	9.1 (.84)	7.9 (1.45)
**Spider**						
Control	.69 (.16)	.26 (.14)	.43 (.17)	.46 (.22)	7.2 (1.33)	6.0 (2.10)
Spider Fear	.68 (.20)	.18 (.07)	.50 (.16)	.40 (.20)	8.1 (1.19)	7.6 (1.49)

Note: Mean hit rates (H), false alarm rates (FA), discrimination index (Pr) and response bias (Br) in percentages and confidence ratings (0 – not confident, 10 – absolutely confident) for each picture category and experimental group. Numbers in parentheses represent SD.

### Confidence ratings

Overall, the analyses of confidence ratings calculated for correctly remembered old and correctly rejected new pictures demonstrated significant effects of Picture Category, F (2, 56) = 70.74, p<.001, η2 = .72, and Picture Category×Group, F (2, 56) = 7.74, p<.01, η2 = .22. Unpleasant pictures received higher confidence ratings than neutral and spider pictures, Picture Category, Fs (1, 28) = 83.31, p<001, η2 = .75. and F (1, 28) = 90.53, p<.001, η2 = .76, respectively. Moreover, neutral pictures were also remembered with higher confidence than spider pictures, Picture Category, F (1, 28) = 42.25, p<.001, η2 = .60. Importantly, confidence ratings were higher for correctly recognized spider pictures in the spider fear than the control group, Group, F (1, 28) = 5.20, p<.05, η2 = .16, see Table 1. No group effects were found for the other stimulus materials Fs (1, 28)<1, ns.

### Event Related Potentials

The ERP waveforms are displayed in Figure 2. Replicating previous studies, correctly recognized old pictures elicited overall more positive ERP amplitudes over centro-parietal scalp areas than correctly rejected new pictures, Memory, F (1, 28) = 22.29, p<.001, η2 = .44, Memory×Picture Category, F (1.634, 56) = 1.50, p = .23, η2 = .05, Memory×Picture Category×Group, F (1.634, 56) = 2.18, p = .13, η2 = .07. For unpleasant and neutral pictures this significant old/new difference, Memory, Fs (1, 28)≥5.5, ps<.05 did not differ between both groups, Memory×Group, Fs (1, 28)<1, ns. In contrast, analyses performed for spider pictures revealed significant differences in the ERP old/new effect between the spider fear and the control group, Memory×Group, F (1, 28) = 7.0, p<.05, η2 = .20. Post-hoc tests showed that old spider pictures prompted an enhanced positivity compared to new pictures in spider-fearful participants, F (1, 14) = 5.01, p<.05, η2 = .26, but not in controls, F (1, 14) = 1.99, p = .18, η2 = .12 (see also bottom panel in Figure 2). Although number of hits and correct rejections were lowest for spider pictures, there were enough trials (average: 16) for this picture category that could be included in waveform analysis. Based on previous studies we also tested for early frontal ERP old/new differences [26] in a 300 to 500 ms time window. We found a significant difference between correctly classified old when compared to new pictures, Memory, F (1, 28) = 11.2, p<.01. However, this early frontally located old/new difference was not modulated by picture content and did not differ between both experimental groups.

Replicating previous ERP studies [1], [49], the P1 was overall more pronounced in spider-fearful than control individuals, Group, F (1, 28) = 4.0, p = 0.5, η2 = .13, Picture Category×Group F (1.848, 56)<1, ns. Moreover, as in earlier studies [1], [33], [35], an increased EPN and LPP was found when viewing unpleasant and spider pictures, compared to neutral material, Picture Category, F (1.408, 56) = 14.50, p<.001, η2 = .34 and Picture Category F (1.634, 56) = 22.76, p<.001, η2 = .45, for the EPN and LPP, respectively. The effects of an enhanced EPN and LPP for spider when compared to neutral pictures were significantly more pronounced in spider fearful when compared to control participants, Picture Category×Group, F (1, 28) = 23.35, p<.001, η2 = 0.44 and Picture Category×Group, F (1, 28) = 6.65, p<.05, η2 = 19, for the EPN and LPP, respectively. Unpleasant and neutral picture comparisons did not reveal any significant group differences, Fs (1, 28)<1, ns. When calculated for each single picture category the EPN and LPP did not differ as a function of group, Fs (1, 28)<1, ns.

## Discussion

In the present study we used behavioral and electrophysiological measures to investigate long-term recognition memory for unpleasant, neutral and spider pictures in individuals with spider fear and non-fearful controls. Replicating previous findings, we found enhanced recognition memory performance accompanied with better confidence ratings for unpleasant when compared to neutral pictures [8], [9], [10], [50]. Memory performance for these materials did not differ between both groups. In the ERPs, enhanced positivity was found for remembered “old” when compared to correctly classified “new” pictures [23], [26]. This ERP old/new effect was observed over centro-parietal scalp areas during the 400–800 ms time interval, indicating recollection-based recognition. Contrary to our expectations, the ERP old-new difference did not differ between neutral and unpleasant pictures as in earlier studies using long retention intervals (e.g., [10], [44], [51]). This lack of interaction was due to the strong old/new effect observed for neutral pictures and not due to a weak old/new effect for unpleasant pictures, both effects are strong and statistically significant. Methodological reasons might account for this deviant pattern of results. In contrast to earlier studies [10], [44], [51], in which pictures were only presented once during encoding, pictures were shown for multiple times in the present study, probably facilitating ERP old-new differences [52], particularly for neutral stimuli. Moreover, a number of neutral pictures were presented in blocks that did not include unpleasant (or spider) pictures. Recent studies found that memory for neutral pictures is better when presented in pure blocks than mixed with emotional stimuli (e.g., [53]–[56]), indicating that neutral pictures in pure blocks may allocate more attentional resources for a detailed analysis than in mixed blocks, resulting in elaborate internal representations and a more pronounced ERP old/new effect for these cues.

One major aim of the current study was to investigate whether we would find different behavioral and/or electrophysiological memory effects for the phobia-relevant material in low and high fear volunteers. In general, our findings indicate that spider pictures were recognized with lower accuracy and confidence in memory than pictures depicting unpleasant and neutral scenes. This seems to be surprising because pictures of spiders are generally more emotional (more arousing and unpleasant) than neutral pictures as indicated by stimulus ratings and electrocortical responses (e.g., [1]) and thus should be better remembered than neutral pictures. On the other hand, given that different spider pictures comprise a very homogenous category where individual exemplars of the category share very similar features, item-similarity between old and new stimuli might have impaired memory performance because spider photographs were perceptually harder to differentiate, even though they included different kinds of spiders displayed with different positions and backgrounds. The poorer discrimination index and the lower confidence ratings for this picture category are in line with this interpretation. Memory accuracy for spider pictures, however, tended to be higher and false alarms were significantly lower in individuals with high spider fear compared to the non-fearful control groups, suggesting that memory performance for these stimuli was better in spider-fearful individuals than in controls. Moreover, participants with spider fear were significantly more confident in their memory decisions for spider pictures than controls indicating a fear-related memory bias. Mirroring this behavioral pattern, significant centro-parietal ERP old-new differences in response to spider pictures were only observed in spider-fearful, but not in control individuals. Because the centro-parietal old-new effect is assumed to reflect recognition memory based on recollection of rich contextual details of the learned event [26], [44], our ERP finding indicates that fear-relevant material was better recollected in spider fearful participants than controls.

Enhanced recollection for fear-relevant material observed in individuals with spider fear when compared to the control group is probably initiated already during the encoding and storage. In fact, the extraction of meaning during the elaborated stimulus processing is supposed to lead to the formation of inter-item associations and enhanced memory consolidation [57]–[59]. Indeed, multiple studies found [1], [33], [34], [35], [60], [61] facilitated processing of fear-related stimuli in individuals with elevated specific fears, as revealed by early and late ERP differences, an effect that was suggested to reflect a state of enhanced attention to feared stimulus materials Such prioritized stimulus processing was also evident during picture viewing at recognition in our study. Enhanced perceptual processing of phobia-related stimuli was also reported in several functional neuroimaging studies showing increased activations in the lateral occipital, posterior parietal, and inferior temporal cortex in specific phobic volunteers during processing of their feared pictures relative to neutral materials [62]–[65]. Confirming the assumption that the facilitated perceptual stimulus processing may be regulated by limbic structures, individuals with small animal phobia also exhibited increased amygdala and insula activations during their feared picture encoding [42], [62], [65], [66]–[69]. A recent PET study performed by Ahs and collaborators [11] demonstrated that these phobic stimuli that prompted stronger amygdala and parahippocampal activations were correlated with better memory for feared stimuli.

In sum, our results indicate that fear-relevant stimuli may be deeply encoded and easily recollected in spider-fearful individuals. Previous studies indicate that the exposure to a phobic stimulus is associated with increased retrieval of fear memories and unpleasant post-event recollection, which results in an enhanced fear response [70]. Unfortunately, these processes might strengthen the phobic response maintaining or even aggravating anxiety symptomatology. Moreover, resulting in an increased avoidance and reinforcing negative beliefs these processes might hinder recovery and interfere with exposure during treatment [16]. Reducing stimulus associated memory retrieval might have potentially beneficial effects on extinction-based psychotherapy. In this context, recent studies focused on promising retrieval-impairing effects of stress and glucocorticoids [71]–[72]. In addition, acute stress induced shortly before extinction was demonstrated to reduce expectancy ratings during the retrieval test that was performed on a subsequent day [73]. This suggests that glucocorticoids might facilitate the consolidation of extinction memory, which may also enhance extinction-based treatment programs [71], [74], [75].

Taken together, the present data indicate enhanced memory processing for phobia-relevant materials in spider-fearful participants. Moreover, we also found that spider-fearful individuals allocate more attentional resources for evaluative processing of phobia-relevant pictures than non-anxious controls. Future research may consider focusing on the relationship between fear memory retrieval, extinction memory and symptom reduction in the course of treatment as well as in the post-treatment phase.
