# Correction: Remembering the Object You Fear: Brain Potentials during Recognition of Spiders in Spider-Fearful Individuals

**DOI:** 10.1371/journal.pone.0134150

**Published:** 2015-07-21

**Authors:** Jaroslaw M. Michalowski, Mathias Weymar, Alfons O. Hamm

In the Funding section, the grant number from the funder National Science Centre is listed incorrectly. The correct grant number is: 2011/03/D/HS6/05951.

## References

[1] Michalowski JM, Weymar M, Hamm AO (2014) Remembering the Object You Fear: Brain Potentials during Recognition of Spiders in Spider-Fearful Individuals. PLoS ONE 9(10): e109537 doi:10.1371/journal.pone.0109537 25296032 10.1371/journal.pone.0109537 PMC4190313

